# Supplementary material for: Rainforest conversion to rubber and oil palm reduces abundance, biomass and diversity of canopy spiders
Source: PeerJ. 2022 Aug 16;10:e13898. doi: 10.7717/peerj.13898 (PMC9390325; doi:10.7717/peerj.13898)
Supplement: Supplemental Information 2 [file peerj-10-13898-s002.pdf]


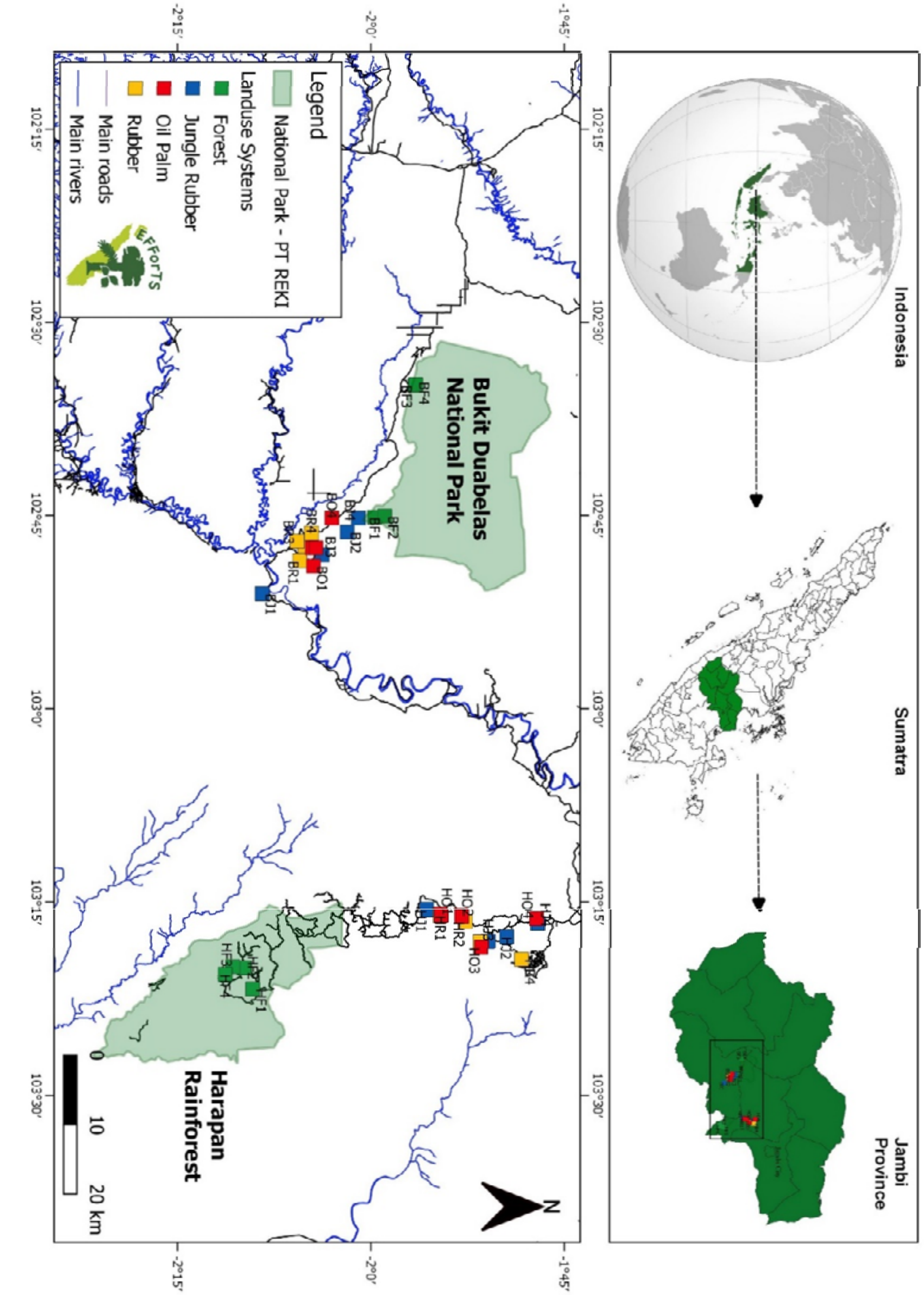

FIGURE S1. Location of the 32 study plots in Jambi Province, Sumatra, Indonesia, arranged in two landscapes near reference sites, i.e. Bukit Duabelas National Park and Harapan Rainforest. Land-use systems are coded by color (green = rainforest, blue = jungle rubber, yellow = rubber, red = oil palm).

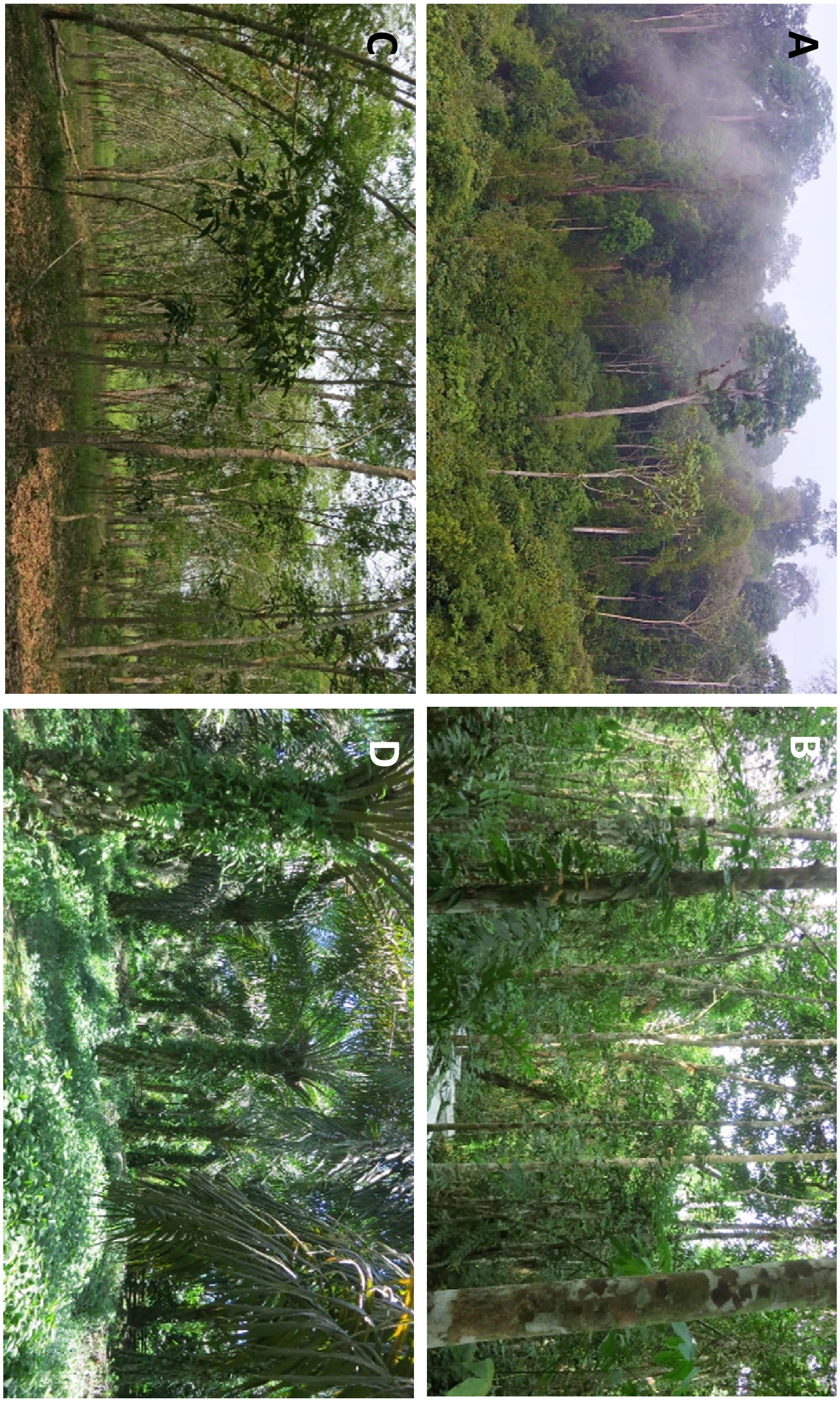

FIGURE S2. Four land-use systems investigated. Lowland rainforest (A), jungle rubber (B), and smallholder monocultures of rubber (C) and oil palm (D). Photos by Mathias Ditscherlein (A) and Jochen Drescher (B-D)

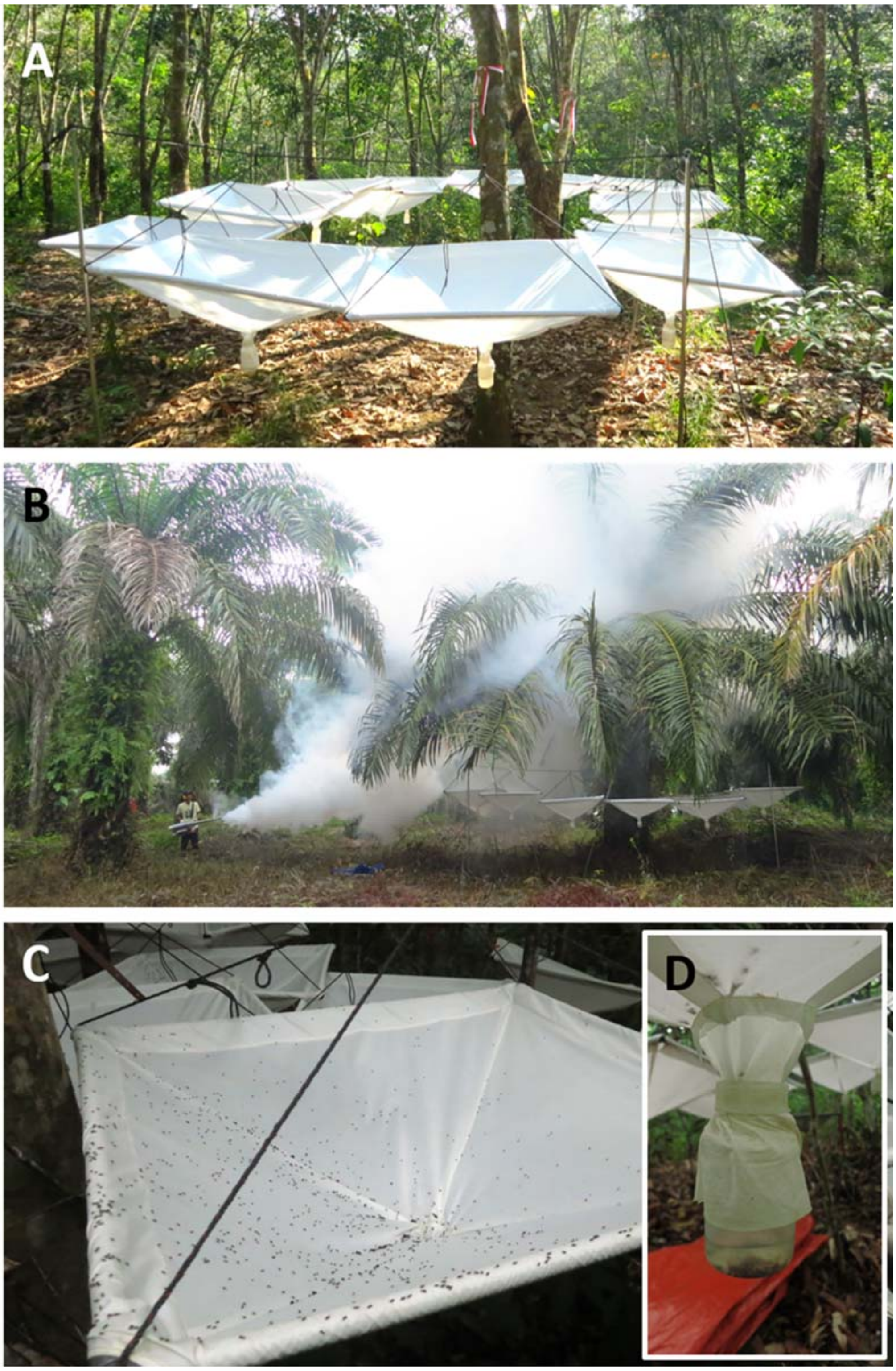

FIGURE S3. Canopy fogging method details: (A) 16 collection traps, each 1 m * 1 m, in smallholder rubber plantation. (B) Canopy fogging in smallholder oil palm, planted 10 years previously. (C) Collecting trap, with stunned insects and spiders. (D) Plastic bottle underneath collection trap, filled with 96% EtOH and arthropods.

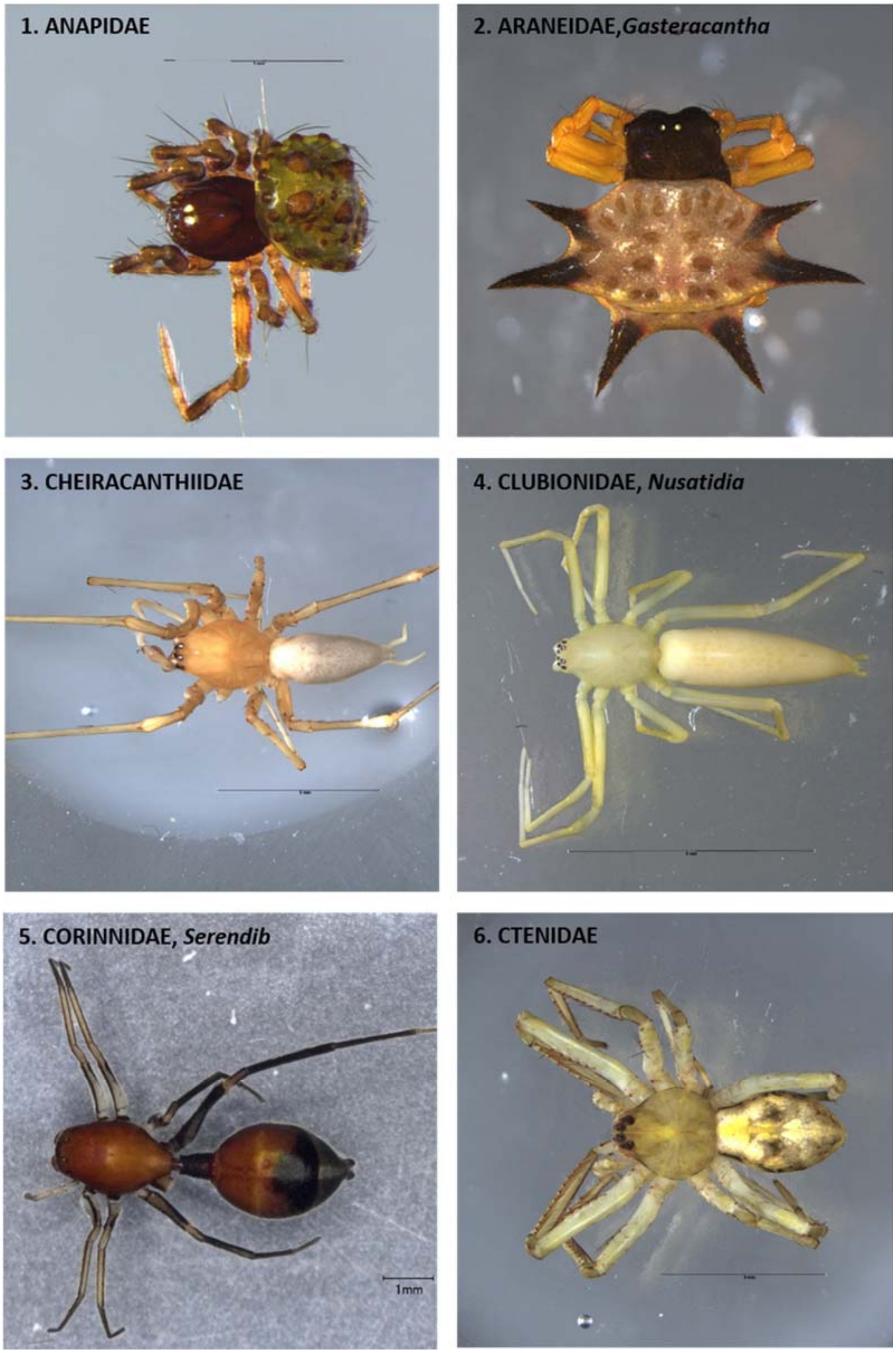

FIGURE S4: 1-6. Canopy spider families collected by canopy fogging in Jambi Province, Sumatra, Indonesia. Family names given in bold, genus names, if known, in italics.

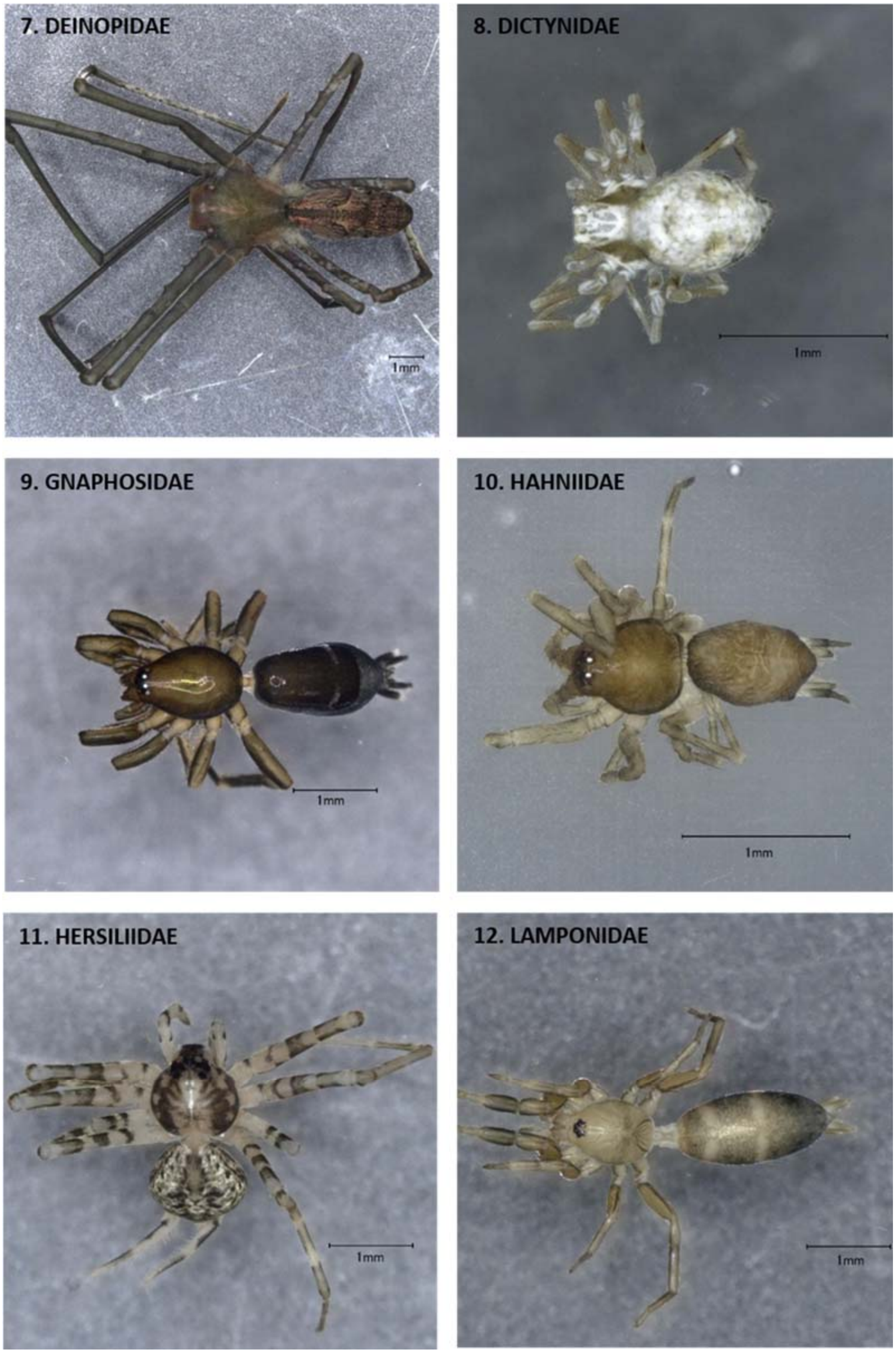

FIGURE S4: 7-12. Canopy spider families collected by canopy fogging in Jambi Province, Sumatra, Indonesia. Family names given in bold, genus names, if known, in italics.

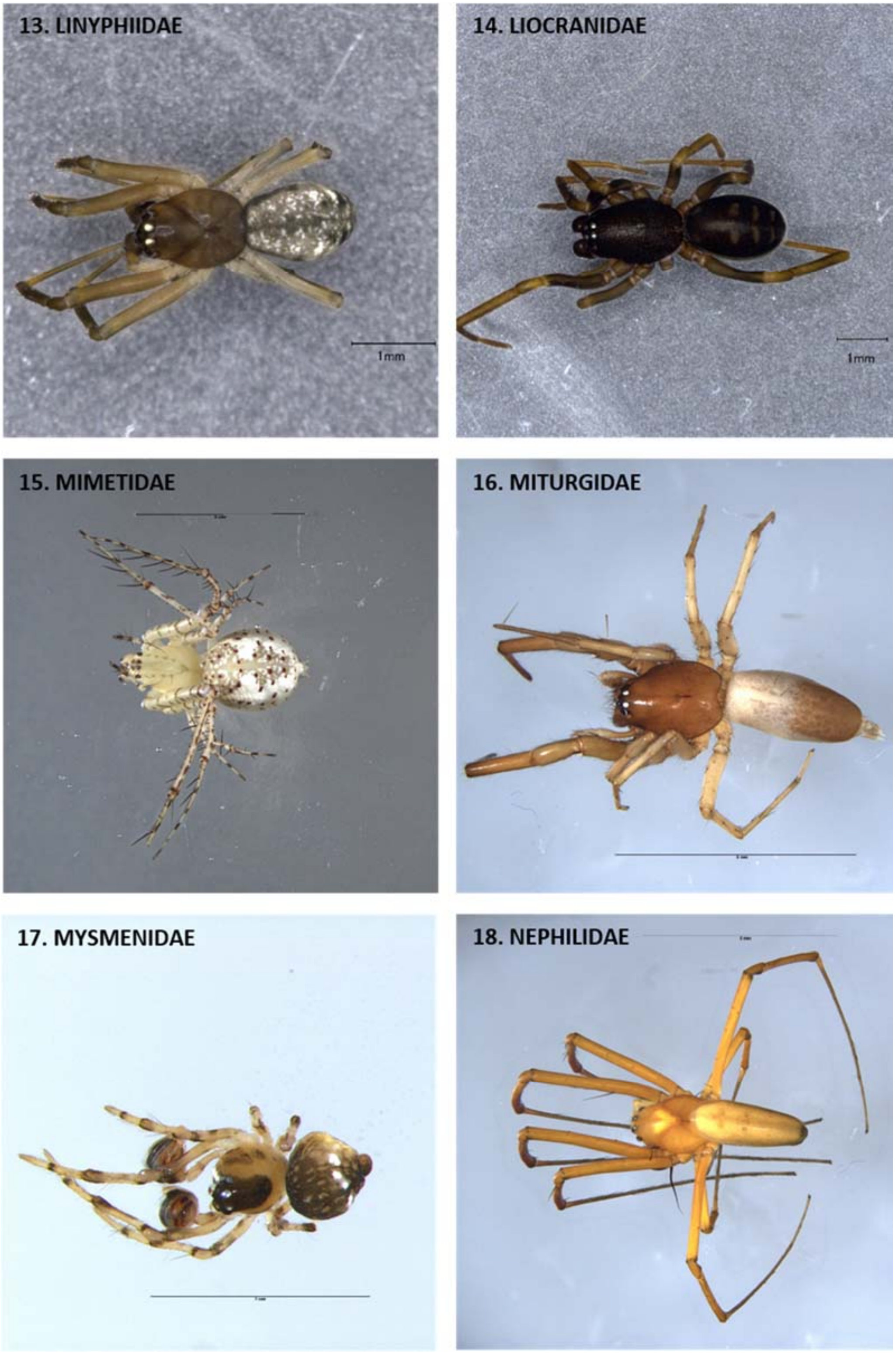

FIGURE S4: 13-18. Canopy spider families collected by canopy fogging in Jambi Province, Sumatra, Indonesia. Family names given in bold, genus names, if known, in italics.

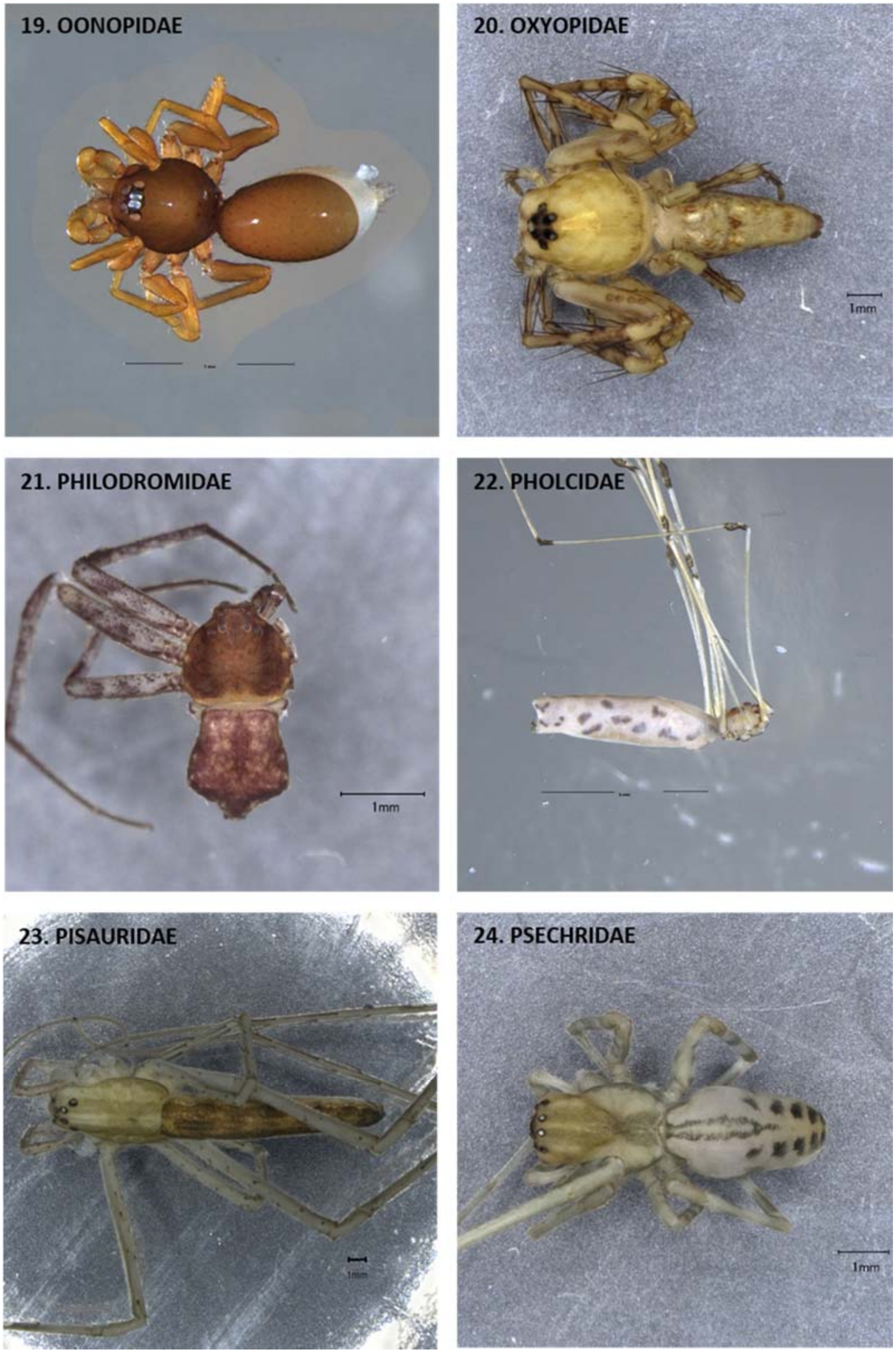

FIGURE S4: 19-24. Canopy spider families collected by canopy fogging in Jambi Province, Sumatra, Indonesia. Family names given in bold, genus names, if known, in italics.

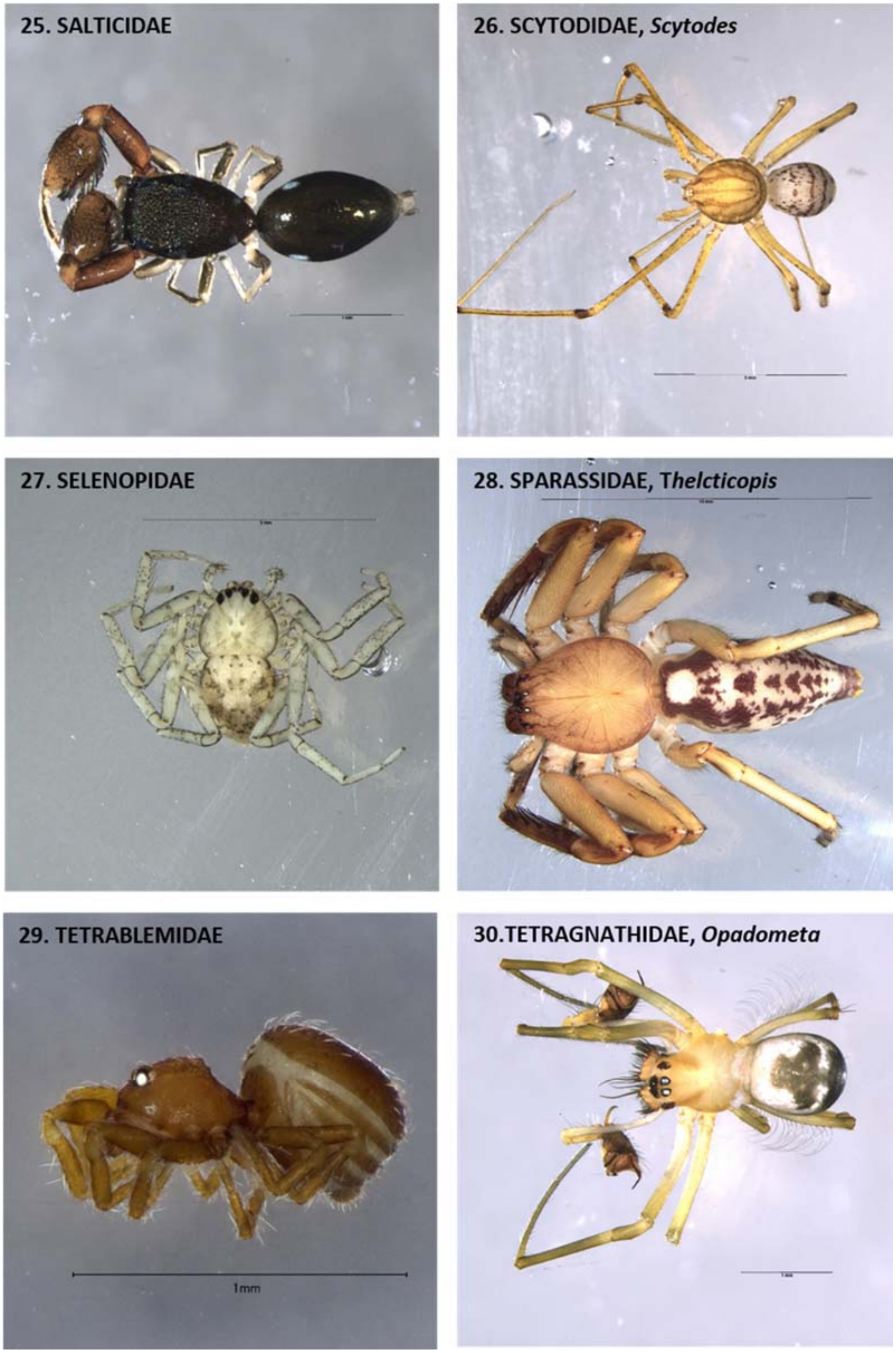

FIGURE S4: 25-30. Canopy spider families collected by canopy fogging in Jambi Province, Sumatra, Indonesia. Family names given in bold, genus names, if known, in italics.

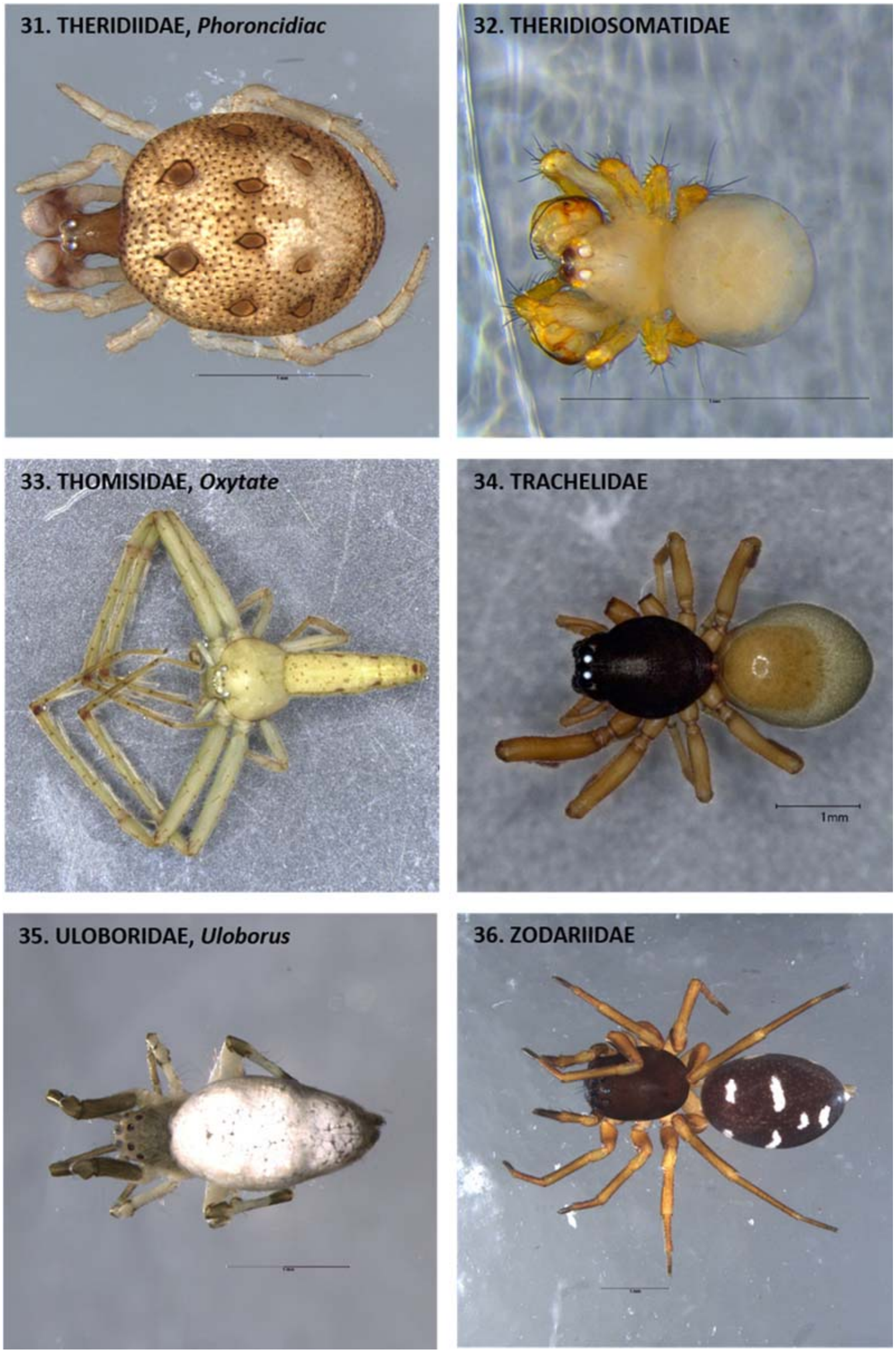

FIGURE S4: 31-36. Canopy spider families collected by canopy fogging in Jambi Province, Sumatra, Indonesia. Family names given in bold, genus names, if known, in italics.

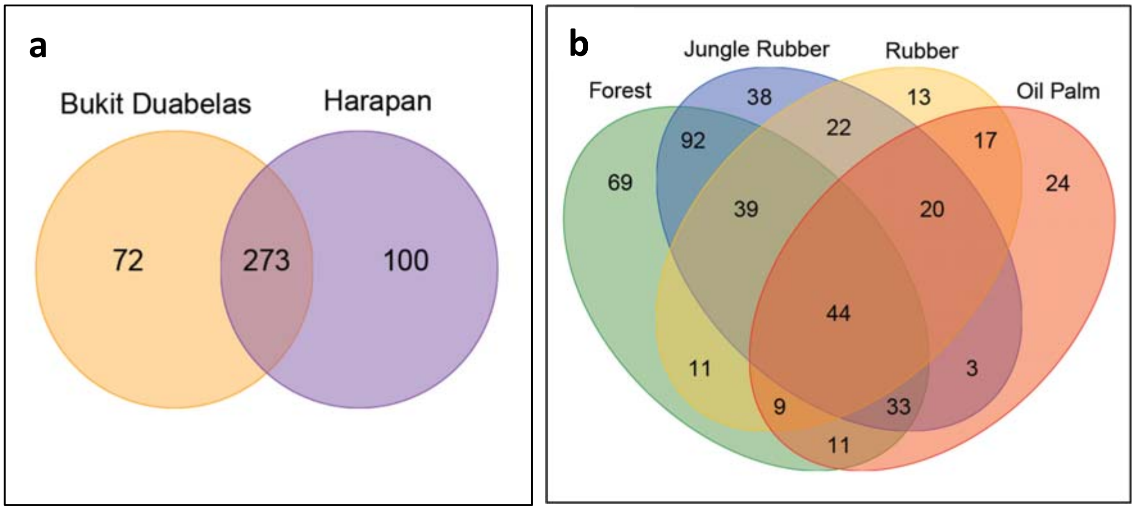

FIGURE S5. Venn diagram of overall canopy spider species overlap between (a) two landscapes (Bukit Duabelas and Harapan) and (b) among four land use systems (rainforest, jungle rubber, rubber, oil palm) in Jambi province, Sumatra, Indonesia.

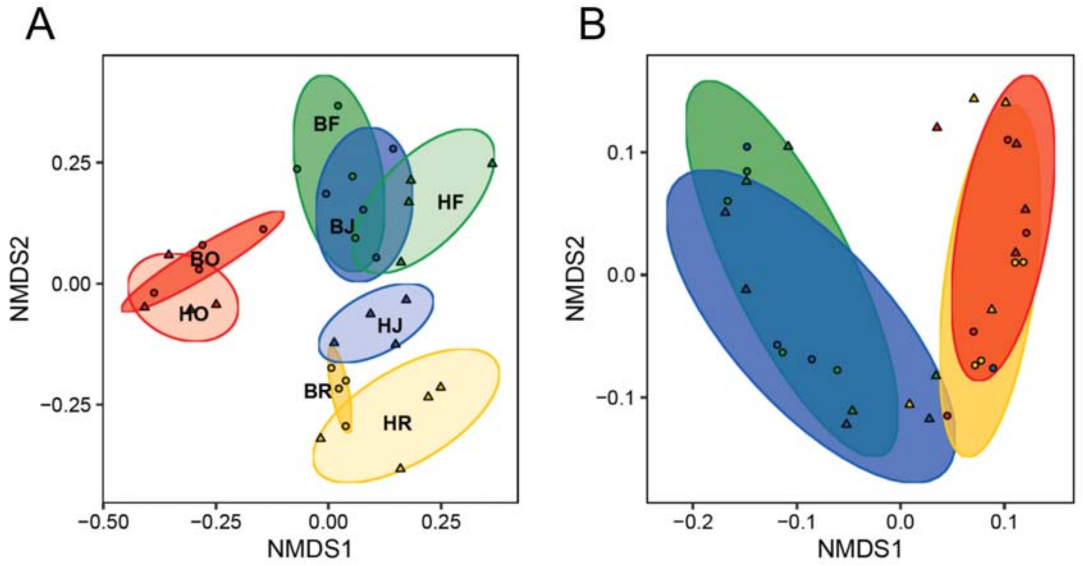

FIGURE S6. NMDS of turnover (A) and nestedness (B) of canopy spider communities in four land-use systems and two landscapes in Jambi, Sumatra, based on Sørensen dissimilarities (F = rainforest, J = jungle rubber, R = rubber, O = oil palm, B = Bukit Duabelas landscape / circles, H = Harapan landscape / triangles). Turnover was influenced by both land use and landscape (interaction: Wilk's λ = 0.18, $F_{3,18}$ = 2.5, P = 0.005; land use: Wilk's λ = 0.01, $F_{3,18}$ = 16.0, P < 0.001; landscape: Wilk's λ = 0.14, $F_{1,6}$ = 18.3, P < 0.001), while differences in nestedness were only driven by land use (Wilk's λ = 0.18, $F_{3,15}$ = 3.2, P < 0.001).

TABLE S1. Eigenvalues of the 36 families of the overall canopy spider community.

family	CCA1	CCA2	CCA3
Anapidae	-0.93266	-0.10610	-0.43759
Araneidae	0.25724	-0.20081	-0.04978
Cheiracanthiidae	0.02836	0.24334	-0.03265
Clubionidae	0.43665	0.23970	-0.13786
Corinnidae	-0.26835	-0.11653	0.05731
Ctenidae	-0.93556	-0.31352	-0.41667
Deinopidae	-1.19780	-0.34653	-0.75539
Dictynidae	-0.07676	-0.38438	0.87916
Gnaphosidae	0.84202	-0.37112	-0.28036
Hahniidae	-0.38854	0.25690	-0.19816
Hersiliidae	-0.39782	0.01280	0.02737
Lamponidae	0.31565	-0.24417	0.71808
Linyphiidae	0.00202	-0.16293	-0.15193
Liocranidae	1.23703	-1.59540	0.70010
Mimetidae	-0.41466	0.03751	0.45101
Miturgidae	0.13382	0.36543	0.32237
Mysmenidae	-0.45189	-0.23269	0.65322
Nephilidae	0.11988	-0.45532	1.32320
Oonopidae	-0.21368	0.14561	0.02208
Oxyopidae	-0.21490	0.21521	0.12310
Philodromidae	0.61637	0.40917	-0.43098
Pholcidae	-0.75123	-0.05447	0.13186
Pisauridae	-0.02812	0.55344	0.14783
Psechridae	-0.78657	0.00454	0.48691
Salticidae	0.21565	-0.09491	0.07669
Scytodidae	-0.35584	0.18303	0.34693
Selenopidae	-1.02968	-0.50566	0.22296
Sparassidae	-0.15920	0.05524	0.08853
Tetrablemidae	-0.68486	0.22278	0.09997
Tetragnathidae	0.57204	-0.44104	0.12718
Theridiidae	-0.38896	-0.17619	-0.21874
Theridiosomatidae	-0.20280	0.67992	0.38185
Thomisidae	0.22405	0.35281	-0.01047
Trachelidae	0.16905	0.07210	-0.09073
Uloboridae	-0.27626	-0.58823	0.29443
Zodariidae	0.10403	0.37265	0.05156

TABLE S2. Eigenvalues of the 445 morphospecies of the overall canopy spider community.

Morphospecies	CCA1	CCA2	Morphospecies	CCA1	CCA2
AraCori009	-1.01037	-1.89848	AraOono003	0.06736	-0.04552
AraSpar010	-1.01037	-1.89848	AraAran041	0.99987	-0.04044
AraThom004	-1.01037	-1.89848	AraTrid092	-0.09655	-0.02948
AraTrid021	-1.01037	-1.89848	AraTrid058	1.15363	-0.02031
AraTrid044	-1.01037	-1.89848	AraTrid086	-0.98935	-0.01866
AraUlob005	-1.00014	-1.76045	AraSpar016	-0.66365	-0.01402
AraSalt067	1.14984	-1.58985	AraTrid082	-1.58156	-0.01304
AraTtra012	1.14984	-1.58985	AraTrid097	-0.53509	-0.00946
AraScyt002	1.17362	-1.57695	AraAran024	0.69042	-0.00631
AraLioc001	1.26019	-1.57024	AraSalt008	-0.09165	-0.00132
AraSalt097	1.26019	-1.57024	AraTrid060	1.09420	0.00671
AraSalt082	1.31408	-1.49405	AraCori011	-0.05565	0.00910
AraSalt065	1.30726	-1.48889	AraPhol009	-1.43137	0.01364
AraUlob001	-0.97968	-1.48437	AraTrid039	-0.10689	0.01509
AraSalt048	1.45890	-1.42220	AraLiny003	1.30199	0.01545
AraAran030	1.35779	-1.40174	AraTrid030	-0.32687	0.01572
AraTtra002	-0.89515	-1.39613	AraTrid023	-0.74682	0.01718
AraSpar004	-0.96945	-1.34634	AraAran008	0.27666	0.01961
AraAran031	1.37380	-1.31520	AraPisa001	-0.76153	0.02461
AraPisa004	1.37380	-1.31520	AraAran057	-0.08649	0.02555
AraThom046	1.37380	-1.31520	AraGnap002	1.38873	0.02616
AraGnap003	1.43071	-1.30352	AraGnap004	0.84450	0.02733
AraTtra005	1.43443	-1.30101	AraTrid029	-1.03662	0.02797
AraTtra007	1.45892	-1.30070	AraAran035	0.59691	0.03366
AraAran027	1.50775	-1.28670	AraTrid007	-0.90785	0.03431
AraUlob007	1.45916	-1.27520	AraSpar023	0.61369	0.04347
AraSalt083	1.36607	-1.24363	AraTrid045	0.69293	0.04986
AraSele001	-1.30279	-1.23646	AraAran012	-0.72432	0.05001
AraSalt006	-0.93012	-1.22662	AraTrid013	-0.21917	0.05364
AraGnap007	1.01820	-1.22538	AraAran047	0.92532	0.06314
AraSalt084	1.18543	-1.18853	AraHers002	-0.14926	0.06387
AraSalt050	1.34959	-1.17565	AraCori015	-0.69029	0.07015
AraAran049	1.55659	-1.15120	AraLiny002	-0.69029	0.07015
AraAran051	1.55659	-1.15120	AraThom010	-0.51808	0.07213
AraClub011	1.55659	-1.15120	AraSalt028	-0.81304	0.07482
AraGnap011	1.55659	-1.15120	AraThom031	-0.66704	0.07629
AraTrid072	1.55659	-1.15120	AraClub012	-0.22613	0.10687
AraAran042	-1.30363	-1.13797	AraSalt004	-0.40682	0.11080
AraTrid004	-0.48322	-1.13782	AraTrid089	-0.49670	0.11327

Morphospecies	CCA1	CCA2	Morphospecies	CCA1	CCA2
AraUlob012	0.87932	-1.13061	AraTrid031	-0.12495	0.11328
AraAran039	-0.67962	-1.11844	AraSalt010	0.16115	0.11362
AraDict001	-0.20063	-1.09701	AraTtra011	0.77297	0.11565
AraSalt055	0.71059	-1.09169	AraSalt001	-0.66103	0.11636
AraOxyo004	-1.15495	-1.08807	AraSalt077	-0.08037	0.13154
AraTrid005	-0.61393	-1.07336	AraCori010	0.25170	0.13626
AraTrid018	-0.94899	-1.07026	AraAran006	-0.63826	0.14046
AraSalt053	1.19282	-1.04653	AraOono007	-0.87996	0.14851
AraSalt074	1.62354	-0.97960	AraAran003	-0.65652	0.15449
AraTrid063	1.62354	-0.97960	AraTble001	-0.65811	0.15454
AraCori006	-0.77076	-0.95053	AraScyt001	-0.38007	0.15573
AraTrid080	0.82153	-0.93524	AraLiny006	-1.07042	0.16293
AraClub021	1.26120	-0.91480	AraThom011	-0.54950	0.16991
AraTrid065	0.27577	-0.90903	AraOxyo006	-0.64382	0.17548
AraSalt051	1.31472	-0.86537	AraThom041	-0.71501	0.19258
AraAran046	1.14742	-0.83872	AraClub017	0.81265	0.19343
AraUlob002	-0.32859	-0.82718	AraHahn004	-0.79027	0.19424
AraSalt002	0.56079	-0.79115	AraTrid093	-0.46769	0.19425
AraSalt022	0.14725	-0.79023	AraTrid095	-0.42999	0.19625
AraAran028	1.33769	-0.78162	AraPhol005	-0.35142	0.19705
AraAran060	0.15361	-0.77573	AraSalt061	0.17462	0.20301
AraAran062	0.15361	-0.77573	AraTrid028	0.00531	0.20407
AraThom040	1.31389	-0.76687	AraLiny004	-0.95066	0.21178
AraSalt080	0.93833	-0.76658	AraSalt023	-0.00513	0.21268
AraAran005	0.87792	-0.74627	AraTrid054	-0.60558	0.21672
AraTtra009	0.67494	-0.73478	AraSalt095	0.97751	0.23869
AraTrid001	-0.47859	-0.73304	AraAran053	1.05750	0.23881
AraSalt009	-0.89047	-0.71133	AraAran010	-0.35343	0.23937
AraLiny001	-0.23110	-0.71021	AraSalt071	-0.86320	0.24812
AraSalt049	1.28086	-0.70921	AraTrid079	-0.92994	0.25069
AraLiny008	-0.64435	-0.69203	AraLiny005	-0.90176	0.25439
AraTrid053	-1.22724	-0.68286	AraClub016	-0.03411	0.26094
AraAran001	-1.20202	-0.64738	AraTsom001	-0.35533	0.26114
AraUlob003	-0.48228	-0.64234	AraSalt068	0.65525	0.26223
AraTrid083	-1.19591	-0.63710	AraAran014	-0.22889	0.26955
AraMysm001	-0.35716	-0.63194	AraCori016	-0.22889	0.26955
AraSpar007	-0.95080	-0.62636	AraTrid098	-0.22889	0.26955
AraTrid047	1.37698	-0.62015	AraGnap006	-0.02522	0.27621
AraPsec002	-0.31095	-0.60305	AraTrid014	-0.11059	0.28118
AraTrid099	-1.34092	-0.58844	AraSalt018	-0.32711	0.28712
AraTrid012	-0.58040	-0.58781	AraTrid052	-0.10414	0.29641
AraTrid026	0.46216	-0.58074	AraCori018	-0.31069	0.29729

Morphospecies	CCA1	CCA2	Morphospecies	CCA1	CCA2
AraDein001	-1.59521	-0.57444	AraThom008	-0.43347	0.30495
AraLiny011	-1.59521	-0.57444	AraTrid043	0.09755	0.30535
AraUlob009	-1.59521	-0.57444	AraTrid050	1.03899	0.31162
AraAran043	0.58865	-0.57161	AraAran040	-0.41020	0.31837
AraLamp001	0.58865	-0.57161	AraOxyo009	-0.08072	0.32719
AraSalt064	0.58865	-0.57161	AraSalt089	-0.08561	0.33572
AraThom033	0.58865	-0.57161	AraTrid062	-0.49541	0.33638
AraThom034	0.58865	-0.57161	AraCten003	-0.04005	0.34068
AraTrid066	0.58865	-0.57161	AraOxyo007	-0.04005	0.34068
AraOono005	0.60873	-0.56781	AraThom028	-0.04005	0.34068
AraSalt086	1.31807	-0.53597	AraClub014	0.66865	0.34294
AraPhol011	-1.22049	-0.52531	AraClub007	-0.05298	0.34882
AraPhol002	-0.67621	-0.52524	AraSalt011	0.35574	0.34923
AraOono002	-0.40335	-0.51603	AraAran058	-1.56623	0.35137
AraAran029	1.40056	-0.51603	AraGnap005	-1.56623	0.35137
AraOxyo012	0.93967	-0.51113	AraPsec001	-1.56623	0.35137
AraSalt003	-0.87385	-0.50675	AraThom036	-1.56623	0.35137
AraSalt058	0.66687	-0.48726	AraTrid096	-1.56623	0.35137
AraSpar008	-1.12342	-0.48310	AraOxyo010	-0.38646	0.35805
AraTtra014	0.76679	-0.47890	AraTrid025	-0.50183	0.36332
AraUlob008	-1.59605	-0.47594	AraSalt026	0.04598	0.36506
AraSalt017	-0.11074	-0.47209	AraAran020	0.38861	0.36674
AraTrid069	-0.82467	-0.46572	AraZoda001	0.02723	0.37090
AraSalt056	0.71177	-0.46251	AraTrid076	-0.31422	0.37122
AraThom009	1.22162	-0.45665	AraGnap009	-0.35874	0.37801
AraTrid008	0.19054	-0.45301	AraSalt066	0.87309	0.37844
AraTrid020	0.50080	-0.45100	AraSalt024	0.66595	0.38056
AraAran015	1.25419	-0.45024	AraChei003	-0.27589	0.38142
AraTrid067	-1.59632	-0.44311	AraSalt087	-1.00341	0.38184
AraTrid061	-1.37003	-0.41499	AraSpar005	-0.30843	0.39268
AraThom006	-0.25466	-0.41424	AraOxyo015	-0.53843	0.40621
AraSalt007	-0.58894	-0.40036	AraThom035	0.23145	0.41576
AraSpar003	0.12432	-0.39554	AraThom002	0.27746	0.42246
AraTrid002	-0.71795	-0.39040	AraTrac001	-0.05028	0.42283
AraClub002	-0.70020	-0.38724	AraOxyo002	-0.02675	0.42681
AraTtra001	0.96667	-0.38553	AraSalt060	0.02582	0.43380
AraClub009	0.74737	-0.38270	AraOxyo011	-0.94808	0.43488
AraSalt054	-0.08247	-0.37875	AraMysm002	1.00109	0.43641
AraSalt032	0.51159	-0.37863	AraThom007	-0.32097	0.46222
AraAran055	-1.59688	-0.37745	AraClub008	0.37857	0.47296
AraCori026	-1.59688	-0.37745	AraAran002	0.29205	0.48055
AraSalt075	-1.59688	-0.37745	AraSalt025	-0.35581	0.48609

Morphospecies	CCA1	CCA2	Morphospecies	CCA1	CCA2
AraThom032	-1.59688	-0.37745	AraSalt092	-0.90170	0.48822
AraThom044	-1.59688	-0.37745	AraClub006	-0.20841	0.49208
AraUlob004	-1.02400	-0.36575	AraThom019	1.00497	0.49581
AraLiny012	-1.44793	-0.36038	AraTrid015	0.36033	0.49636
AraPhol010	-1.44793	-0.36038	AraSalt088	0.38553	0.49732
AraOono008	0.57220	-0.35981	AraSalt085	-0.56764	0.50060
AraNeph001	0.69733	-0.35502	AraUlob006	1.26890	0.50434
AraPhol003	-0.91007	-0.35423	AraClub018	-0.27833	0.51442
AraSpar019	-1.00469	-0.35410	AraClub020	-0.27833	0.51442
AraCori004	-0.83355	-0.34204	AraOxyo016	-0.27833	0.51442
AraThom001	-0.78802	-0.33557	AraAran056	0.05143	0.51664
AraTtra015	-0.80713	-0.33511	AraClub010	0.21631	0.51775
AraHers001	-0.69002	-0.33376	AraSalt079	0.21631	0.51775
AraSalt073	0.92021	-0.32670	AraSalt081	0.21631	0.51775
AraAran021	1.28086	-0.32208	AraTrid074	0.21631	0.51775
AraNeph003	1.08772	-0.31502	AraThom037	0.02174	0.52930
AraSpar009	0.15492	-0.31047	AraMitu001	0.42535	0.56428
AraClub025	1.17055	-0.30853	AraThom021	0.21180	0.56838
AraLiny010	1.17055	-0.30853	AraPhol007	-0.20024	0.57585
AraSalt070	1.17055	-0.30853	AraSalt069	0.05774	0.57621
AraTrid075	1.17055	-0.30853	AraTrid070	0.25436	0.58088
AraMime001	-0.53930	-0.30314	AraThom005	-0.39329	0.59107
AraAran048	-1.37387	-0.30260	AraSalt043	0.19420	0.59803
AraAran044	-0.51978	-0.30258	AraPhil002	0.00951	0.59844
AraAran025	0.82404	-0.30120	AraPisa003	-0.28273	0.59913
AraCori017	-0.14448	-0.29790	AraOxyo014	-0.55448	0.60314
AraTtra003	-1.06418	-0.29757	AraThom043	-0.27384	0.60540
AraSalt062	1.13255	-0.29649	AraSalt040	0.70141	0.61020
AraTrid038	-0.45390	-0.29338	AraChei004	0.27339	0.61244
AraOxyo005	-0.45308	-0.28697	AraSalt090	0.27339	0.61244
AraMime002	-0.29733	-0.28467	AraTrid078	0.27339	0.61244
AraAran045	-0.32421	-0.28052	AraClub004	0.90946	0.61506
AraSpar002	-0.41543	-0.27139	AraSalt047	0.48306	0.62084
AraCori012	0.43936	-0.27006	AraThom038	-0.57522	0.63599
AraTrid048	0.73997	-0.26888	AraTrid084	0.43468	0.64391
AraHahn001	-0.27787	-0.26367	AraOxyo013	-0.20241	0.66256
AraTrid024	-0.15548	-0.25758	AraTrid087	-0.08666	0.66590
AraOxyo001	-0.46862	-0.25403	AraAnap002	-0.89143	0.67323
AraOxyo008	-1.37428	-0.25335	AraAran061	-0.81837	0.69404
AraAnap001	0.11758	-0.25135	AraLiny009	-0.20753	0.69845
AraCori014	0.11758	-0.25135	AraGnap001	1.14813	0.72088
AraTrid034	0.11758	-0.25135	AraTrid035	0.27094	0.72340

Morphospecies	CCA1	CCA2	Morphospecies	CCA1	CCA2
AraClub024	0.41134	-0.24378	AraSpar018	0.06165	0.72461
AraAran004	-1.23141	-0.24284	AraSalt072	0.53656	0.73059
AraClub003	0.47772	-0.23946	AraClub023	0.66543	0.74651
AraTrid056	-1.59075	-0.23169	AraTrid027	-0.25772	0.75016
AraChei002	0.81205	-0.23130	AraSpar021	0.70593	0.75035
AraCori027	-0.93249	-0.22418	AraThom027	-0.15693	0.76020
AraLiny007	0.87326	-0.22109	AraTtra013	-0.69266	0.76335
AraPhil001	0.00943	-0.21889	AraSalt046	0.88981	0.77494
AraTrid091	-1.24039	-0.21829	AraSalt021	-0.20464	0.77848
AraTrid077	-1.30008	-0.21199	AraCori021	-0.10733	0.78038
AraTrid090	-1.21505	-0.19285	AraThom015	0.96200	0.78570
AraSalt078	0.33415	-0.19173	AraAran063	0.08034	0.78858
AraThom029	0.53279	-0.19010	AraThom025	0.04287	0.80165
AraSalt005	-0.66131	-0.18959	AraSalt052	0.39936	0.80627
AraSalt091	-0.09137	-0.18513	AraSalt063	0.63854	0.80939
AraAran038	-1.00037	-0.18379	AraTtra006	1.13521	0.81629
AraTrid068	0.96246	-0.17953	AraThom026	-0.29429	0.81648
AraTrid011	-0.03216	-0.17539	AraClub001	0.73353	0.81946
AraTrid033	-0.96138	-0.17402	AraSalt037	-0.05242	0.82431
AraOono006	-0.72860	-0.16995	AraSpar022	0.46089	0.84116
AraClub022	-0.15044	-0.16932	AraAran023	0.57138	0.84388
AraSalt027	0.38231	-0.16465	AraPhol006	0.16516	0.84597
AraSpar001	-0.10333	-0.15578	AraSpar013	0.27665	0.84788
AraTrac003	0.27856	-0.15229	AraSalt076	0.61564	0.89871
AraUlob010	0.17988	-0.15103	AraClub013	-0.20866	0.92418
AraTrid010	-0.73240	-0.14381	AraClub019	0.43051	0.92646
AraSalt019	-0.32800	-0.14380	AraSpar011	0.67994	0.93109
AraThom016	0.95249	-0.14059	AraAran054	0.27582	0.94637
AraCori025	-1.58666	-0.13451	AraCori024	-0.25588	0.96934
AraTrid006	-1.19209	-0.13042	AraHahn003	-0.25588	0.96934
AraTrid064	0.72886	-0.12932	AraSpar014	-0.25588	0.96934
AraClub015	-1.15169	-0.12926	AraSpar015	-0.25588	0.96934
AraCten002	-1.15169	-0.12926	AraThom045	-0.25588	0.96934
AraSalt093	-1.15169	-0.12926	AraClub005	0.89875	0.97472
AraSalt094	-1.15169	-0.12926	AraThom018	0.23747	1.02098
AraTrid094	-1.15169	-0.12926	AraSalt029	0.56867	1.02365
AraUlob013	-1.15169	-0.12926	AraSalt014	0.95225	1.02428
AraPhol004	-0.51818	-0.12275	AraSpar017	0.79522	1.03396
AraCten001	-0.49186	-0.12006	AraGnap010	0.64562	1.04227
AraThom030	0.71415	-0.10496	AraPhil003	0.62699	1.05010
AraTrid055	0.90830	-0.10100	AraThom042	1.26425	1.05152
AraPisa002	0.17944	-0.09944	AraTrid081	1.26425	1.05152

**Morphospecies**	**CCA1**	**CCA2**	**Morphospecies**	**CCA1**	**CCA2**
AraCori003	-0.34454	-0.09939	AraTrid085	0.25377	1.06341
AraPhol001	-0.40252	-0.09420	AraSalt059	-0.06210	1.07892
AraSpar020	0.21087	-0.08193	AraAran037	-0.65171	1.10569
AraPhol008	-0.82935	-0.07982	AraSalt099	-0.65171	1.10569
AraTrid051	1.22333	-0.07932	AraSalt045	0.94534	1.14935
AraSalt039	-0.55747	-0.07817	AraSpar012	0.73008	1.16017
AraSalt030	0.00209	-0.07771	AraAran016	0.59136	1.17623
AraThom012	0.01545	-0.07573	AraTrid049	1.12588	1.23362
AraCori005	-0.48406	-0.06507	AraSalt015	1.22305	1.25558
AraTrid059	-1.12239	-0.06132	AraSalt042	1.32022	1.27754
AraTrid003	-0.40855	-0.05672	AraSalt044	1.32022	1.27754
AraTtra004	-0.58350	-0.04992	AraThom039	-0.20130	1.35951
AraOono001	-0.45112	-0.04823	AraAran034	-0.50884	1.42543
AraSalt034	-0.36171	-0.04762	AraThom017	1.10662	1.64355
AraChei001	-0.44021	-0.04669	AraSalt098	-0.63531	1.65348
AraTrac002	0.05720	-0.04600			
